# Diabetic retinopathy at diagnosis of type 2 diabetes in Scotland

**DOI:** 10.1007/s00125-012-2596-z

**Published:** 2012-06-12

**Authors:** H. C. Looker, S. O. Nyangoma, D. Cromie, J. A. Olson, G. P. Leese, M. Black, J. Doig, N. Lee, R. S. Lindsay, J. A. McKnight, A. D. Morris, S. Philip, N. Sattar, S. H. Wild, H. M. Colhoun, on behalf of the Scottish Diabetic Retinopathy Screening Collaborative and the Scottish Diabetes Research Network Epidemiology Group

**Affiliations:** 1 Medical Research Institute, University of Dundee, The Mackenzie Building, Kirsty Semple Way, Dundee, DD2 4BF UK; 2 Department of Public Health, NHS Lanarkshire, Glasgow, UK; 3 Eye Unit, Aberdeen Royal Infirmary, Aberdeen, UK; 4 Diabetic Retinopathy Screening Centre, Inverness, UK; 5 Diabetes Unit, Forth Valley Royal Hospital, Larbert, UK; 6 Scottish Diabetic Retinopathy System, Inverness, UK; 7 Institute of Cardiovascular and Medical Sciences, University of Glasgow, Glasgow, UK; 8 Metabolic Unit, Western General Hospital, Edinburgh, UK; 9 Grampian Diabetes Research Unit, Aberdeen, UK; 10 Centre for Population Health Studies, University of Edinburgh, Edinburgh, UK

**Keywords:** Diabetic retinopathy, Diabetic retinopathy screening, Scotland, Type 2 diabetes

## Abstract

**Aims/hypothesis:**

The aim of this study was to examine the prevalence of and risk factors for diabetic retinopathy in people with newly diagnosed type 2 diabetes mellitus, using Scottish national data.

**Methods:**

We identified individuals diagnosed with type 2 diabetes mellitus in Scotland between January 2005 and May 2008 using data from the national diabetes database. We calculated the prevalence of retinopathy and ORs for risk factors associated with retinopathy at first screening.

**Results:**

Of the 51,526 people with newly diagnosed type 2 diabetes mellitus identified, 91.4% had been screened by 31 December 2010. The median time to first screening was 315 days (interquartile range [IQR] 111–607 days), but by 2008 the median was 83 days (IQR 51–135 days). The prevalence at first screening of any retinopathy was 19.3%, and for referable retinopathy it was 1.9%. For individuals screened after a year the prevalence of any retinopathy was 20.5% and referable retinopathy was 2.3%. Any retinopathy at screening was associated with male sex (OR 1.19, 95% CI 1.14, 1.25), HbA_1c_ (OR 1.07, 95% CI 1.06, 1.08 per 1% [11 mmol/mol] increase), systolic BP (OR 1.06, 95% CI 1.05, 1.08 per 10 mmHg increase), time to screening (OR for screening >1 year post diagnosis = 1.12, 95% CI 1.07, 1.17) and obesity (OR 0.87, 95% CI 0.82, 0.93) in multivariate analysis.

**Conclusions/interpretation:**

The prevalence of retinopathy at first screening is lower than in previous UK studies, consistent with earlier diagnosis of diabetes. Most newly diagnosed type 2 diabetic patients in Scotland are screened within an acceptable interval and the prevalence of referable disease is low, even in those with delayed screening.

## Introduction

Diabetic retinopathy is one of the leading preventable causes of visual impairment in the UK [1]. Treatment can prevent vision loss, but requires early detection and careful monitoring to be most effective [2]. The prevalence of diabetic retinopathy at diagnosis of type 2 diabetes is a useful indirect measure of how well a healthcare system is performing with respect to diabetes detection; where type 2 diabetes is present for a long time prior to diagnosis prevalence rates of diabetic retinopathy at diagnosis will be high [3]. Prevalence at diagnosis also indicates to what extent there is an urgency to perform retinal screening after diagnosis. Finally, understanding the characteristics of those patients with type 2 diabetes who have diabetic retinopathy at diagnosis is of practical use for targeting of screening. The aim of this study was to examine the prevalence and determinants of diabetic retinopathy among people with newly diagnosed type 2 diabetes in Scotland (population 5.1 million). We also assess the coverage, uptake and rapidity of retinal screening delivery in this population.

## Methods

The data used were from an anonymised extract of the Scottish Care Information – Diabetes Collaboration (SCI-DC), a clinical database that holds data on people diagnosed with diabetes in Scotland. The SCI-DC database was rolled out across Scotland from 2000 and the estimated coverage of the total diabetic population is around 99%. SCI-DC captures key diabetes-related data items, such as BMI, HbA_1c_, lipids and BP, from all hospitals and 1,100 general practices in Scotland. SCI-DC data were linked to death records held by the National Records of Scotland using probabilistic linkage.

The national roll out of the Scottish Diabetic Retinopathy Screening (DRS) programme to improve the availability of high-quality retinal screening in Scotland [4] began in 2006, attaining nationwide coverage by January 2007. All eligible patients (aged 12 years and over) registered on the SCI-DC are invited to participate in this programme. All new registrants are automatically entered as new patients on the DRS database, which triggers the appointment process. Those who are already attending eye clinics for diabetic eye disease, those declining screening and those who are too unfit or frail for screening are suspended from the programme, with their status reviewed at least every 3 years. The retinal examination involves a single-field digital photograph, with mydriasis if required, with centralised grading [5] or, when photographic images are ungradable, slit-lamp examination. Slit-lamp examination gradings were not available for all health boards for the whole period of the study, but were included for analysis when available. The use of a single central-field digital photograph for the detection of sight-threatening retinopathy has been validated [6–8]. The programme includes quality-control protocols to ensure the quality of the photographs and the grading [9].

Each eye is given a retinopathy and a maculopathy grade. The subsequent action taken is determined by the most severe finding in the worst eye. The grading scheme for the DRS programme is shown in Table 1. Visual acuity is often unaffected during the early stages of diabetic retinopathy, but may deteriorate as the severity of the retinopathy and maculopathy worsens with proliferative retinopathy (R4) and referable maculopathy (M2), both of which are sight-threatening conditions. Previous analyses from the pilot phase of the DRS programme estimate the prevalence of diabetic retinopathy at 20% [10] and the referral rate for eye disease at 3% [5]. The most recent data for the years 2009–2010 indicate a stable referral rate of 3.5% [9].

**Table 1:** Grading scheme of the Scottish Diabetic Retinopathy Screening Collaboration

Grade	Explanation/description
Retinopathy	
R0	No diabetic retinopathy
R1 (mild)	BDR—mild
	•At least one dot haemorrhage or microaneurysm with or without hard exudates
R2 (moderate)	BDR—moderate
	•Four or more blot haemorrhages (i.e. ≥Airlie House standard photograph 2a) in one hemi-field only (inferior and superior hemi-fields delineated by a line passing through the centre of the fovea and optic disc)
R3 (severe)	BDR—severe
	•Any of the following features:
	–Four or more blot haemorrhages (i.e. ≥Airlie House standard photograph 2a) in both inferior and superior hemi-fields
	–Venous beading (≥Airlie House standard photograph 6a)
	–IRMA (≥Airlie House standard photograph 8a)
R4 (proliferative)	PDR
	•Any of the following features
	–New vessels
	–Vitreous haemorrhage
Maculopathy	
M1 (observable)	•Lesions within a radius of >1 but <−2 disc diameters of the centre of the fovea
	•Any hard exudates
M2 (referable)	•Lesions within a radius of <−1 disc diameter of the centre of the fovea
	•Any blot haemorrhages
	•Any hard exudates

Adapted from Scottish Diabetic Retinopathy Grading Scheme [34]

BDR, background diabetic retinopathy; IRMA, intraretinal microvascular abnormalities; PDR, proliferative diabetic retinopathy

For this analysis, the retinopathy/maculopathy grade for an individual was defined as the grade of the worst eye. We extracted data on all those registered on SCI-DC with type 2 diabetes diagnosed between 1 January 2005 and 31 May 2008 (the most recently available capture of new patients). DRS data for this cohort were available up to the end of 2010, as were data for other covariates including sex, age, BMI, HbA_1c_, BP, total cholesterol and socioeconomic status (as assessed by the Scottish Index of Multiple Deprivation [SIMD], a measure indexed by residence [11]). For covariates, the measurement used was the one made at the time nearest to the diagnosis of type 2 diabetes. When no measure was available within 180 days of diagnosis, the data were considered to be missing; the exception was BMI, for which data were considered missing when no observation was available within 365 days of diagnosis. For individuals diagnosed prior to the launch of the DRS programme, the time to screening was calculated as the time from diagnosis to the first SCI-DC entry representing retinal examination, regardless of the source; for those diagnosed after the launch of the DRS programme, the time to screening was calculated as the time to first DRS screening.

The primary date of type 2 diabetes diagnosis was based on the date entered into the SCI-DC by clinicians; if multiple dates were entered we took the earliest date. This date was checked against multiple data sources including prescription and hospital discharge records for any prior evidence of type 2 diabetes. We excluded 2,406 individuals (4.4% of potentially eligible individuals) where there was significant discrepancy over the date of diagnosis (i.e. a difference in date of diagnosis of >120 days). Where there was a discrepancy of <120 days we selected the earliest date for our analyses. Diabetes type was determined according to type recorded in SCI-DC with the addition of an algorithm to identify individuals at high risk of being mislabelled based on age of diagnosis and early use of insulin.

Approval was obtained from the Scotland A Research Ethics Committee, the Caldicott (data privacy) Guardian for the 14 Scottish Health Boards and the ISD Privacy Advisory Committee.

### Statistical analysis

We used *t* tests to compare continuous variables and *χ* ^2^ tests to compare dichotomous variables among people screened within 1 year of diagnosis vs those screened later, and people who had successful screening vs those with ungradable photographic images. Logistic regression was used to examine independent associations of variables with the prevalence of retinopathy at screening. Cox proportional hazards models were used to examine independent associations of variables with time from diagnosis to first retinal screening. To control for observed clustering of times to first screening, associated with the year of diagnosis (i.e. the strong relationship between time to screening and year of diagnosis), we fitted a multivariate mixed-effects Cox proportional hazards model, with year of diagnosis taken as a random effect, and age, sex and any of the variables that were significantly associated in univariate analysis entered as fixed effects. All statistical analysis was undertaken using the R statistical package [12].

## Results

There were 51,526 people with newly diagnosed type 2 diabetes eligible for the study. Over half were male (*n* = 28,576 [55%]) and the mean age at diagnosis was 61.8 years (SD 12.8 years). As of 31 December 2010, 47,090 (91.4%) people had attended a retinal screening examination, with 25,322 (53.8% of the screened population) screened within 1 year of diagnosis. A total of 4,436 (8.6%) had not attended a DRS screening (Table 2). The leading reason for not being screened related to ill health, with 2,143 (4.2%) dying prior to the end of 2010. (Among those who died before screening, the median time from diabetes diagnosis to death was 375 days, interquartile range [IQR] 169–657 days.) Suspension from the programme because of eye clinic attendance affected only 0.8% of the population. The proportion of unscreened individuals declined over time: of all individuals diagnosed in 2005, 1,917 (12.1%) had not been screened as of 31 December 2010, while for subsequent years those numbers fell to 1,283 (10.1%) in 2006, 941 (8.2%) in 2007 and 238 (5.4%) in 2008.

**Table 2:** Screening the newly diagnosed type 2 population

Retinopathy screening status	Number (%) of newly diagnosed patients with type 2 diabetes (*n* = 51,526)
Died before screening	2,143 (4.1)
Already under the care of eye clinic/retinal screening outside the DRS system	399 (0.8)
Unscreened for other reasons (including choice not to enter screening programme, poor health or no longer resident in Scotland)	1,894 (3.7)
Total not screened before end 2010	4,436 (8.6%)
Ungradable images with no slit-lamp examination data	3,567 (6.9)
At least one graded screening result available	43,523 (84.5)
Total screened before end 2010	47,090 (91.4)

Complete covariate data for the period around diagnosis of type 2 diabetes were available for the majority of individuals with a record of screening (*n* = 40,194, 85.4%). The proportions of missing data by variable were: 8.5% for HbA_1c_ (*n* = 4,006); 6.3% for total cholesterol (*n* = 2,981); 6.0% for BMI (*n* = 2,832); 5.2% for BP (*n* = 2,470); and 0.6% for SIMD (*n* = 293).

### Time to screening

**Fig. 1:**
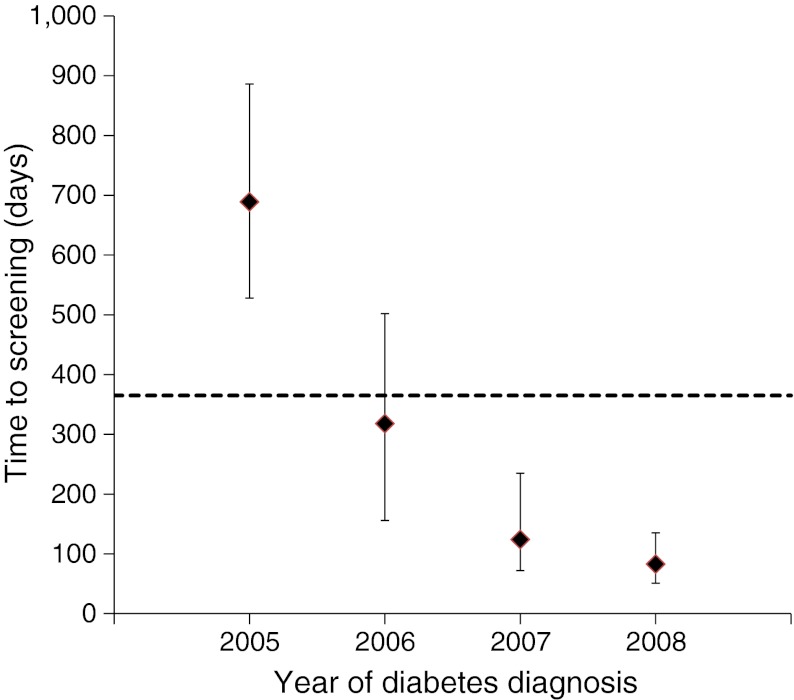
Median time to retinal screening from diagnosis of type 2 diabetes in days, by year of diabetes diagnosis. Error bars indicate the 25th to 75th percentiles; dotted line indicates 1 year

Of the 47,090 who were screened, the median time to first retinal screening was 315 days (IQR 111–607 days). However, time to first screening was strongly related to year of diagnosis (Fig. 1); individuals diagnosed in 2005 had a median time to any documented screening of almost 18 months (median = 540 days, IQR 258–747) falling to <3 months (median = 83 days, IQR 51–135) in 2008. In a mixed-effects multivariate Cox proportional hazards model, with year of diagnosis treated as a random effect and the other variables as fixed effects, male sex (HR 1.03, 95% CI 1.01, 1.05), older age (HR 1.01, 95% CI 1.00, 1.02 per 10 years of age), systolic BP ≥135 mmHg (HR 1.07, 95% CI 1.04, 1.10), diastolic BP ≥80 mmHg (HR 1.03, 95% CI 1.01, 1.04), total cholesterol ≥4.5 mmol/l (HR 1.05, 95% CI 1.03, 1.07) and lower socioeconomic status (HR 1.02, 95% CI 1.00, 1.04) were all statistically significantly associated with longer time to screening, while obesity was associated with a shorter time to screening (HR 0.97, 95% CI 0.95, 0.99) within a multivariate model (*p* < 0.05). HbA_1c_ at diagnosis was not associated with time to screening.

### Prevalence of retinopathy and maculopathy

The prevalence of any diabetic retinopathy at first screening was 19.3% and that of referable diabetic retinopathy was 1.9% (Table 3), with only 0.7% having R3 or R4 grade retinopathy.

**Table 3:** Prevalence of retinopathy at first screening for all people successfully screened

Finding	Frequency, *n* (%) (*n* = 43,523)
No eye disease	35,114 (80.7)
R0 and no maculopathy (M0)	35,114 (80.7)
Non-referable eye disease	7,568 (17.4)
R1 and no maculopathy (M0)	7,341 (16.9)
R2 and no maculopathy (M0)	39 (0.1)
R0 or R1 or R2 with non-referable maculopathy (M1)	188 (0.4)
Referable eye disease	841 (1.9)
R0 or R1 or R2 with referable maculopathy (M2)	523 (1.2)
R3 ± any maculopathy	190 (0.4)
R4 ± any maculopathy	128 (0.3)

The prevalence of diabetic retinopathy varied by time to screening; for individuals screened within 1 year of diagnosis (*n* = 25,322) the prevalence of any diabetic retinopathy was 18.3% and 1.6% for referable diabetic retinopathy vs 20.5% and 2.3%, respectively, for people screened more than a year after diagnosis (*p* < 0.0001 for both comparisons). The prevalence was highest for those first screened >2 years after diagnosis (*n* = 7,512) who had a prevalence of any diabetic retinopathy of 20.7% and of referable diabetic retinopathy of 2.7%. Individuals screened within 3 months of diagnosis (*n* = 9,354) had a prevalence of any diabetic retinopathy of 18.5% and referable diabetic retinopathy of 1.4%.

Details of the diagnosis for the eye disease causing follow-up with the eye clinics were not available for 0.8% of this population. If we assume all these people (*n* = 399 [see Table 2]) are attending an eye clinic because of diabetic retinopathy, then the upper limit of any diabetic retinopathy for the population is 20.0% and 2.6% for referable diabetic retinopathy.

### Ungradable images (R6)

Not all screening examinations resulted in gradable images. When the DRS programme does not obtain satisfactory photographs, slit-lamp examinations are undertaken. However, results from these examinations have not routinely been entered into the central database until recently. Within the current analyses 578 individuals had results available from slit-lamp examinations and are categorised according to the retinopathy status found by slit-lamp examination. Overall, 7.6% of those screened during the study period had an ungradable image and no slit-lamp examination result available. Individuals with ungradable images were older (mean age 72 years), had higher systolic BP (140.7 mmHg), lower diastolic BP (mean 77.8 mmHg), lower total cholesterol (mean 4.99 mmol/l), lower HbA_1c_ (7.9% [63 mmol/mol]), and lower BMI (mean 30.1 kg/m^2^) when compared with those who had successful screening (*p* < 0.001 for all differences using the *t* test).

Of the 3,567 people with ungradable images at their first screening, 2,198 (61.6%) had no diabetic retinopathy at their next screening, with 356 (10.0%) having evidence of diabetic retinopathy, while the remaining 1,013 (28.4%) had persistently ungradable images. If we assume that this subsequent finding of referable diabetic retinopathy had been present at the first examination and that persistently ungraded eyes all represent diabetic retinopathy then the overall rate of diabetic retinopathy at first screening in the study would increase from 19.3% to 19.9%.

### Risk factors associated with early diabetic retinopathy

In univariate logistic regression models the following variables were associated with the presence of retinopathy at first screening: male sex, lower BMI, higher HbA_1c_, longer time to first retinopathy screening, lower socioeconomic status and higher systolic and diastolic BP. There was no association with age at diagnosis or total cholesterol (data not shown). In a logistic regression model that included all the variables associated with retinopathy in the univariate analyses, together with age, the factors independently associated with retinopathy were male sex, lower BMI, higher HbA_1c_, higher systolic BP and longer time to first retinopathy screening (Table 4). When those not screened because of eye clinic attendance were included in the above model as having retinopathy these risk factor relationships did not change appreciably (data not shown).

**Table 4:** Characteristics near to diagnosis of diabetes mellitus by subsequent retinopathy status

	All (*n* = 47,090)	No diabetic retinopathy (*n* = 35,114)	Diabetic retinopathy (*n* = 8,409)	OR for diabetic retinopathy vs no diabetic retinopathy (95% CI)	*p* value
Male sex	26,341 (55.9%)	19,654 (56%)	5,103 (60.7%)	1.19 (1.14, 1.25)	<0.001
Age (years)	61.3 ± 12.4	60.4 ± 12.0	60.6 ± 12.1	1.02 (0.99, 1.04)	0.163
BMI (kg/m²)	32.0 ± 6.4	32.2 ± 6.4	31.7 ± 6.4	0.87 (0.82, 0.93)	<0.001
HbA_1c_ (%)	8.1 ± 2.1	8.0 ± 2.1	8.4 ± 2.2	1.07 (1.06, 1.08)	0.001
HbA_1c_ (mmol/mol)	65.0 ± 23.1	63.9 ± 23.1	68.3 ± 24.2	1.06 (1.05, 1.08)	<0.001
Systolic BP (mmHg)	139.9 ± 86.8	139.5 ± 99.6	141.1 ± 24.1		
Diastolic BP (mmHg)	80.9 ± 12	80.9 ± 12.2	81.8 ± 11.4	1.01 (0.98, 1.03)	0.572
Higher socioeconomic status	21,308 (45.2%)	15,993 (45.5%)	3,704 (44.0%)	0.96 (0.91, 1.01)	0.122
Median time to screening (days)	315 (111–607)	305 (109–601)	353 (116–625)	1.12 (1.07–1.17)	<0.001

Data are mean ± SD, median with IQR or frequency with percentage

ORs and *p* values were computed by multiple logistic regression with a model that included all variables

ORs for continuous variables are per ten units except for: HbA_1c_, which is given per 1% unit (11 mmol/mol); BMI, which is presented for obese vs non-obese; and time to screening, which is presented for screened after 1 year vs screened within 1 year

## Discussion

Diabetic retinopathy remains a major complication of type 2 diabetes and requires early detection for best treatment [2]. The DRS programme had screened 91.4% of all people in Scotland newly diagnosed with type 2 diabetes by 31 December 2010. The median time from diabetes diagnosis to retinopathy screening declined throughout the study period, with participants diagnosed in 2008 having a median wait to screening of 83 days. The prevalence of any diabetic retinopathy among people with newly diagnosed type 2 diabetes was 19.3%, which is almost half the prevalence of any diabetic retinopathy, 35–39%, reported by the UKPDS [13].

The major strengths of the current study are its use of national-level data, which include the results of retinal photography screening for diabetic retinopathy and covariate data from the time of type 2 diabetes diagnosis. The DRS is the only form of diabetic retinopathy screening recognised for primary care payments in Scotland, so it is the dominant method of diabetic retinopathy screening. Scotland also has a means for linking an individual’s medical data from a variety of sources via a unique medical identifier, which allows the incorporation of data from many sources. The richness of the data sources allowed us to use a variety of data to determine diabetes type. This meant we were able to exclude individuals from the study who showed strong evidence for having type 1 diabetes even if originally classified as having type 2 diabetes. Similarly, we could interrogate a number of data sources to ensure that the individuals included in the study had a consistent date of diagnosis.

There are also weaknesses of this study to consider. The DRS programme is aimed at detecting sight-threatening diabetic retinopathy and relies on a single-field photograph per eye, as has been validated as a means for identifying sight-threatening diabetic retinopathy [6–8]. This approach is less sensitive than the seven-fields-per-eye approach used in the Wisconsin Epidemiologic Study of Diabetic Retinopathy [14] and will miss mild disease, such as peripheral microaneurysms. We also lack data for the presence of diabetic retinopathy among the small proportion of individuals in Scotland who obtain their screening outside the DRS programme. This is primarily via ophthalmology clinics and, as <1% of the population attend such screenings, even if we assumed all of these individuals had diabetic retinopathy it would not make a major difference to our prevalence estimate (20.0% vs 19.3%).

In the UKPDS, which recruited patients with new-onset type 2 diabetes aged 25–65 between 1983 and 1991, the prevalence of any diabetic retinopathy was 35% in women and 39% in men [13]. The UKPDS excluded individuals with severe diabetic retinopathy at baseline. Our data are not directly comparable as the UKPDS used four fields and so was more likely to detect early grades of peripheral diabetic retinopathy than our study. However, much has changed since the UKPDS, including the diagnostic criteria for diabetes as well as health policy in the UK. There is now a greater emphasis on screening for type 2 diabetes. Unlike the NHS in England and Wales, the NHS in Scotland has not adopted systematic screening for type 2 diabetes [15]; however, risk profiling for cardiovascular disease, including testing for type 2 diabetes, has been encouraged [16]. Identifying obese patients who are at high risk for type 2 diabetes is also included in the Quality and Outcomes Framework, a series of standards for primary care practices that provides additional funds on the basis of meeting specific targets [17]. The lower prevalence of diabetic retinopathy at diabetes diagnosis reported in the current study suggests that these system-wide changes have reduced delays in diabetes diagnosis.

Our findings are in line with reports from recent population studies in which the prevalence of diabetic retinopathy ranged from 6% to 23% [3, 14, 18–21]. The lowest estimates (6.2% in Australia [18] and 10.2% in the USA [20]) come from studies that undertook simultaneous diabetes diagnosis and retinal screening. Diabetic retinopathy also occurs in non-diabetic populations [22, 23], with estimates ranging from 5.2% in the Pima Indians [21] to 8% in the general US population [24]. In Australia the prevalence of diabetic retinopathy was 5.8% for those with normal glucose tolerance and 6.7% in people with impaired glucose tolerance or impaired fasting glucose [18]. This suggests that even with moves to minimise delay in diagnosis of diabetes through diabetes screening programmes we would not anticipate achieving a prevalence of diabetic retinopathy at the time of diagnosis of less than 5%. It also raises the question of whether factors other than dysglycaemia may be relevant to the development of diabetic retinopathy in some individuals.

The importance of glycaemia, blood pressure and diabetes duration as risk factors for diabetic retinopathy is already well established [13, 14, 25–27]. Male sex has also been reported as a risk factor in other studies [27]. Results concerning the relationship between BMI and risk for diabetic retinopathy are inconsistent, with both positive [26, 28] and negative associations [29–31] reported. We have not reported the associations with smoking or triacylglycerols because there were insufficient data for individuals in the study at the time of diagnosis.

Of the risk factors for delays in screening found in the current study only high systolic BP was also associated with the presence of diabetic retinopathy at first screening and none of the factors measured had a clinically significant impact on delays in screening. While delays in screening are a concern, the knowledge that the prevalence of diabetic retinopathy and sight-threatening diabetic retinopathy is low even for those screened after 24 months is reassuring. The USA has now started diagnosing diabetes based on the presence of an elevated HbA_1c_ [32]. It is unknown how HbA_1c_ criteria will impact on time to diagnosis in the population and the net effect could be earlier diagnosis because of greater ease in carrying out the test (i.e. no requirement for fasting or glucose challenge), or later diagnosis as HbA_1c_ diagnosis detects fewer individuals than the standard glucose tolerance tests [33]. Our data suggest that a delay in diagnosis of up to 2 years would have a minimal impact on the prevalence of sight-threatening diabetic retinopathy.

## Conclusions

The nationwide DRS programme has successfully screened over 90% of individuals with newly diagnosed type 2 diabetes in Scotland, with the majority being screened within 12 months of diagnosis. Delays in screening have become less common over time, indicating improvements in the system as the DRS programme attained full national coverage, with a current median time to screening of <3 months.

When diabetic retinopathy screening was within 3 months of diabetes diagnosis, the prevalence of any diabetic retinopathy was 18.5% and 1.4% for referable diabetic retinopathy. While these prevalences are much lower than those reported in the past they remain higher than estimates from population screening, suggesting that there is still room for earlier diagnosis of type 2 diabetes in this population. However, even among individuals not screened until after a year of diagnosis the prevalence of referable eye disease remains very low.

## Abbreviations

DRS: Scottish Diabetic Retinopathy Screening
IQR: Interquartile range
SCI-DC: Scottish Care Information – Diabetes Collaboration
SIMD: Scottish Index of Multiple Deprivation
